# Psoriasis is associated with increased risk of other autoimmune diseases: A retrospective case-control study using the All of Us research database

**DOI:** 10.1016/j.jdin.2024.03.018

**Published:** 2024-05-09

**Authors:** Priya Marella, Shari R. Lipner

**Affiliations:** a Department of Biological Sciences, New Jersey Institute of Technology, Newark, New Jersey; b Department of Dermatology, Weill Cornell Medicine, New York, New York

**Keywords:** autoimmune disease, comorbidities, psoriasis, risk factors, skin disease

*To the Editor:* Psoriasis is a common chronic inflammatory disease with well-established comorbidities, including cardiovascular disease, and metabolic syndrome.^1^ While a link between psoriasis and autoimmune diseases has been suggested, it is challenging to substantiate associations with uncommon diseases. Therefore, we aimed to examine these associations with a nested matched case-control approach using a large national database.

The *All of Us* database was queried 8/31/23 to 9/13/23 for psoriasis cases, and age, sex, and race matched controls (1:3). Comorbidity frequencies were recorded for psoriasis and control patients. Wald test compared groups (*P* < .05) (Supplementary Methods, available via Mendeley at https://data.mendeley.com/datasets/8rvkx2gkf3/1).

A total of 7478 psoriasis cases and 22,434 matched controls were analyzed, with similar demographics between groups (all *P* > .05, Supplementary Table I, available via Mendeley at https://data.mendeley.com/datasets/8rvkx2gkf3/1). There were correlations between psoriasis and well-established comorbidities, as well as autoimmune diseases, including autoimmune thyroiditis (3.9%, odds ratio [OR] 3.64 [3.06-4.32]), Crohn’s disease (3.9%, OR 5.37 [4.43-6.52]), systemic lupus erythematosus (3.0%, OR 3.45 [2.85-4.19]), uveitis (2.8%, OR 3.69 [3.02-4.52]), lichen sclerosus (1.5%, OR 3.59 [2.72-4.73]), vitiligo (1.2%, OR 4.62 [3.34-6.39]), and alopecia areata (0.9%, OR 3.85 [2.67-5.56]) (Table I). Adalimumab, was the most frequently used tumor necrosis factor alpha (TNF-α) inhibitor amongst psoriasis patients with Crohn’s disease (34.7%), uveitis (22.3%), systemic lupus erythematosus (14.4%), autoimmune thyroiditis (11.7%), and lichen sclerosus (10%). Janus Kinase (JAK) inhibitors, including tofacitinib, were rarely used among psoriasis patients with alopecia areata (6.2%) and vitiligo (1.1%) (Table II).

**Table I:** Comorbidity frequency of patients with psoriasis vs control

	Patients with psoriasis (*n* = 7478)	Case controls (*n* = 22,434)	Odds ratio	*P* value
Codiagnoses, *n* (%)				
Cardiovascular disease	6447 (86.2)	10,012 (44.6)	7.76 [7.23-8.33]	<2.2 × 10ˆ-16
Crohn’s disease	288 (3.9)	166 (0.7)	5.37 [4.43-6.52]	<2.2 × 10ˆ-16
Vitiligo	93 (1.2)	61 (0.3)	4.62 [3.34-6.39]	<2.2 × 10ˆ-16
Hyperlipidemia	4967 (66.4)	7084 (31.6)	4.29 [4.05-4.53]	<2.2 × 10ˆ-16
Hypertension	4790 (64.1)	7054 (31.4)	3.89 [3.68-4.11]	<2.2 × 10ˆ-16
Alopecia areata	65 (0.9)	51 (0.2)	3.85 [2.67-5.56]	<2.2 × 10ˆ-16
Obesity	3279 (43.8)	3899 (17.4)	3.71 [3.51-3.93]	<2.2 × 10ˆ-16
Uveitis	211 (2.8)	175 (0.8)	3.69 [3.02-4.52]	<2.2 × 10ˆ-16
Autoimmune thyroiditis	290 (3.9)	246 (1.1)	3.64 [3.06-4.32]	<2.2 × 10ˆ-16
Lichen sclerosus	110 (1.5)	93 (0.41)	3.59 [2.72-4.73]	<2.2 × 10ˆ-16
Systemic lupus erythematosus	222 (3.0)	197 (0.88)	3.45 [2.85-4.19]	<2.2 × 10ˆ-16
Diabetes mellitus	2353 (31.5)	3092 (13.8)	2.87 [2.70-3.06]	<2.2 × 10ˆ-16

**Table II:** Frequency of biologic use of patients with psoriasis with comorbidities

Comorbidity	Number of cases with comorbidity and psoriasis	Type of medication	Medication use, *n* (%)
Autoimmune thyroiditis	290	Adalimumab	34 (11.7)
		Etanercept	23 (7.9)
		Infliximab	12 (4.1)
		Certolizumab pegol	3 (1.0)
		Golimumab	2 (0.69)
Crohn’s disease	288	Adalimumab	100 (34.7)
		Infliximab	72 (25)
		Certolizumab pegol	45 (15.6)
		Etanercept	14 (4.9)
		Golimumab	4 (1.4)
Systemic lupus erythematosus	222	Adalimumab	32 (14.4)
		Etanercept	22 (9.9)
		Infliximab	13 (5.9)
		Certolizumab pegol	2 (0.9)
		Golimumab	2 (0.9)
Uveitis	211	Adalimumab	47 (22.3)
		Etanercept	31 (14.7)
		Infliximab	17 (8.1)
		Certolizumab pegol	7 (3.3)
		Golimumab	7 (3.3)
Lichen sclerosus	110	Adalimumab	11 (10)
		Etanercept	7 (6.4)
		Infliximab	2 (1.8)
Vitiligo	93	Ruxolitinib	1 (1.1)
		Tofacitinib	1 (1.1)
Alopecia areata	65	Tofacitinib	4 (6.2)

Our study corroborates associations of psoriasis with several autoimmune diseases, with risk similar to that of diabetes mellitus, obesity, hypertension, and hyperlipidemia. In a population-based study of 20,851 patients, psoriasis was associated with increased odds of vitiligo (OR 1.19 [1.01-1.40]), which increased risk of cardiovascular disease (OR 2.00 [1.34–2.97]) and hypertension (OR 2.47 [1.90–3.21]).^2^ A meta-analysis reported an association between alopecia areata and psoriasis (OR 2.71 [2.29-3.21]) and psoriasis with alopecia areata (OR 3.52 [1.27-9.74]).^3^ Psoriasis, autoimmune thyroiditis, Crohn’s disease, uveitis, and lichen sclerosus have shared pathogenesis involving the T helper 17 cells pathway, with increased levels of TNF-α, and anti-TNF-α therapy reducing T helper 17 cell differentiation and inflammatory cytokines.^4^ The Janus kinase-signal transducers and activators of transcription signaling pathway is involved in the pathogenesis of psoriasis, vitiligo and alopecia areata, and therefore JAK inhibitors, may treat these diseases.^5^ A significant proportion of psoriasis patients with Crohn’s disease and uveitis, but a smaller proportion with systemic lupus erythematosus and autoimmune thyroiditis were prescribed TNF-α inhibitors. Only a small percentage of psoriasis patients with vitiligo and alopecia were prescribed JAK inhibitors and a small percentage of patients with lichen sclerosus were given TNF-α inhibitors. Understanding shared disease pathways between psoriasis and other autoimmune diseases may expedite diagnosis of psoriasis comorbidities and lead to improved treatment outcomes and quality of life.

Limitations include retrospective design, miscoding, lack of information on disease severity, and lack of the ability to control for confounding variables including socioeconomic status.

Our study shows a strong association between psoriasis and autoimmune diseases. A review of systems and complete dermatological examination may expedite diagnosis of psoriasis comorbidities, with referral to ophthalmology or gastroenterology for ocular or gastrointestinal symptoms, respectively. TNF-α inhibitors and JAK inhibitors may be underutilized in psoriasis patients with comorbidities with shared pathogenesis.

## Conflicts of interest

Dr Lipner has served as a consultant for Moberg Pharmaceuticals, Eli Lilly, BelleTorus Corporation, and Ortho-Dermatologics. Author Marella has no conflicts of interest to declare.
